# Successful Management of Palatal Developmental Groove–Associated Periodontal Defect Using Palatal Access Flap and Odontoradiculoplasty: A Case Report

**DOI:** 10.1155/crid/5562567

**Published:** 2025-01-17

**Authors:** Nicola Alberto Valente, Giulia Bardini

**Affiliations:** ^1^Department of Surgical Sciences, Division of Periodontology, School of Dental Medicine, University of Cagliari, Cagliari, Italy; ^2^College of Dentistry, AUIB-American University of Iraq Baghdad, Baghdad, Iraq; ^3^Department of Surgical Sciences, Division of Conservative Dentistry and Endodontics, School of Dental Medicine, University of Cagliari, Cagliari, Italy

**Keywords:** case report, developmental groove, nonsurgical treatment, odontoplasty, periodontitis

## Abstract

This case report discusses the successful management of a deep palatal developmental groove associated with Stage III generalized Grade C periodontitis. Despite prior nonsurgical periodontal therapy, the disease progressed rapidly, necessitating further intervention. A comprehensive evaluation revealed generalized periodontitis with localized tooth-related predisposing factor due to a developmental groove in the vital upper left lateral incisor. The initial nonsurgical treatment involved scaling and root planing (SRP) coupled with systemic antibiotics. Significant improvement was observed, except for the site with the developmental groove. Surgical intervention was performed using a palatal access flap odontoplasty and radiculoplasty to eliminate the remaining pocket and enhance plaque control. The procedure successfully resolved the condition, with soft tissue healing observed at the 6-month follow-up with a residual probing depth of 3 mm. This case highlights the challenges associated with palatal developmental grooves as localized aggravating factors in periodontitis. The use of surgical techniques like odontoplasty and flap access proved effective in managing periodontal defects associated with developmental grooves, showcasing a successful outcome in this patient.

## 1. Introduction

While bacterial biofilm, commonly known as plaque, is recognized as the primary, if not the sole, etiological factor, periodontitis can be influenced by various risk factors and conditions that contribute to its onset and progression [1–3]. These conditions can promote the accumulation and formation of plaque, undisturbed by routine home oral hygiene practices, thus acting as facilitators for the primary etiological agent [4].

In the new classification of periodontal and peri-implant diseases and conditions, under the category “other conditions affecting the periodontium,” we find “prostheses and tooth-related factors that modify or predispose to plaque-induced gingival diseases/periodontitis” commonly referred to as “plaque-retentive factors” [3, 4]. These definitions encompass all prosthetic or dental elements that, by promoting plaque accumulation or invading supracrestal tissue attachment, cause sustained inflammation and potential tissue resorption. Tooth-related factors often involve elements or anatomical characteristics of the tooth itself, making plaque control challenging. Among these, the developmental groove is undoubtedly a condition that frequently has negative effects on the periodontium.

The developmental groove typically originates from the tooth's cingulum and extends apically along the crown, involving the root to varying lengths depending on the cases, potentially reaching the apex [5]. It can have variable depths, making it not always easily detectable. It seems not to be such a rare finding, with reported incidences ranging from 2.8% to 8.5% [6, 7], which may be due to differences in diagnostic criteria, research methods, ethnic backgrounds, and geographical locations [6–13].

In most cases, it occurs in maxillary lateral incisors [7], with an incidence of 5.6% reported on these dental elements [9].

The cause of the palatogingival groove remains controversial, although it is generally assumed to originate from an alteration in the development of the internal enamel epithelium and Hertwig's epithelial sheath [14] and is thought to represent a failed attempt to form a second root [15]. Additional etiologic hypotheses have been proposed, including an altered genetic mechanism (genetic factors), or it may occur as a variant of dens invaginatus [16, 17].

A straightforward yet clear classification of developmental grooves divides them into mild or moderate, depending on the extension along the root, when they do not involve the pulp chamber, or complex when they do involve the pulp chamber and extend along the whole length of the root [18]. The complexity referred to in this classification clearly pertains to treatment management since, indeed, those with pulp involvement are theoretically more complex to manage endodontically. Although primarily associated with deep periodontal pockets and alveolar bone loss, pulp necrosis frequently occurs, leading to combined periodontal-endodontic lesions. Therefore, early diagnosis of the palatal groove and the subsequent elimination of the groove in the cervical portion to block bacterial channels are critical for successful treatment. These measures are essential in preventing the formation of progressive periodontal defects and pulp necrosis.

In the past, tooth extraction was the common approach, but now, there are numerous treatment options for PGGs. These include curettage, saucerization, groove sealing up to the cementoenamel junction, endodontic and periodontal interventions, combined endodontic-periodontal procedures, and surgical approaches such as guided tissue regeneration (GTR) and intentional replantation [16].

However, teeth with developmental grooves without pulp involvement can also cause significant problems, especially when the groove is deep and extends into the root, causing undisturbed plaque accumulation and consequent destruction of the periodontium, leading to pocket formation and bone resorption.

Intraoral radiograph represents a noninvasive and cost-effective two-dimensional radiographic imaging technique. It is the primary choice for several purposes. However, this conventional X-ray provides less detailed, overlapping images with inherent geometric distortions, potentially affecting the accuracy and analysis of dental structures. In contrast, dental cone-beam computed tomographic (CBCT) imaging has improved the diagnostic process and overcome the limitations associated with two-dimensional radiography [19, 20]. In the case of combined endodontic-periodontic lesions, CBCT imaging may help the visualization of the communication between the pulp and the periodontium and provide a clear understanding of the depth and length of the palatal groove and the internal anatomy of the root canal [21].

In this case report, we demonstrate the successful management of a deep developmental groove, not involving the pulp and partially extending into the root of the upper left lateral incisor, using access flap and odontoplasty after nonsurgical periodontal therapy in a patient with Stage III generalized Grade C periodontitis, that is, periodontitis with attachment loss greater than 5 mm but without loss of more than four teeth, likely requiring surgical treatment and characterized by rapid progression.

## 2. Case Presentation

A 30-year-old nonsmoking patient, with no significant medical history and in overall good health, presented to our clinic with the following chief complaint: “My gums bleed when I brush, and they are sore. I have also recently noticed pus discharge around the lower right canine.” The patient reported being aware of having periodontitis for 3 years and had undergone a session of scaling and root planing (SRP) at the time of the initial diagnosis and 6 months before coming to our attention at the predoctoral clinic of the dental school, with limited improvement. He also mentioned that the progression of the disease in these few previous years had been quite fast, a fact confirmed by indirect evidence in the radiographic analysis considering his age. Finally, he reported a family history of periodontitis on his mother's side, who had unspecified periodontal health-related issues.

**Figure 1:**
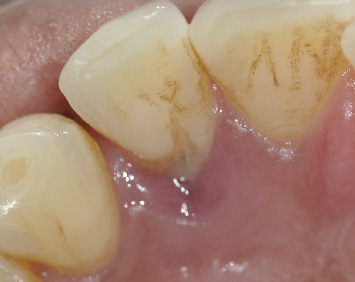
Palatal groove at the first visit with visible suppuration.

**Figure 2:**
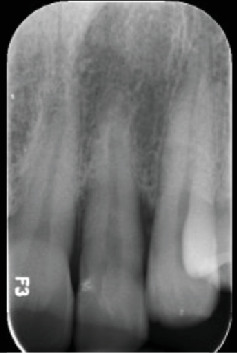
Radiographic image of the tooth revealing normal periradicular tissues with intact lamina dura surrounding the root and a uniform PDL space.

The patient reported brushing two or three times a day with a medium-bristled manual toothbrush and flossing. The intraoral examination did not show caries or other pathologies affecting the teeth or oral mucosa. The patient had a decent oral hygiene, confirmed by a plaque index of 16% according to O'Leary, Drake, and Naylor [22]. Additionally, the patient reported being a bruxist, confirmed by the detection of occlusal surfaces with a certain degree of wear. Intraoral examination revealed a visible developmental groove on the upper left lateral incisor, associated with a 7-mm pocket and pus discharge where the groove was deepening apically (Figure 1). The pulp sensitivity tests exhibited a positive response on Tooth #2.2. Periradicular tests (percussion and palpation) yielded negative results and radiographic examination revealed normal periradicular tissues with intact lamina dura surrounding the root and a uniform PDL space (Figure 2).

Furthermore, the patient had generalized brownish extrinsic pigmentation due to extensive tea consumption. The periodontal chart showed a severe loss of attachment, with several interproximal sites with interproximal attachment loss (iCAL) greater than 5 mm and pockets between 6 and 8 mm associated with extensive bleeding on probing (BOP) equal to 75% of the sites. The lower right canine was confirmed with pus discharge upon sulcular probing. The diagnosis was definitively generalized Stage III Grade C periodontitis, with vital pulp and normal periradicular tissue on Tooth #2.2, thus indicating a pure periodontal lesion, potentially caused by a palatogingival groove.

The initial treatment plan included, along with oral hygiene instructions, supragingival and subgingival plaque control through prophylaxis with ultrasonic scaling and manual SRP, as well as subgingival irrigation with 0.12% chlorhexidine, all performed in a single session. This was accompanied by systemic administration of amoxicillin + metronidazole 500 mg + 250 mg three times a day for 7 days, starting on the same day as the SRP.

**Figure 3:**
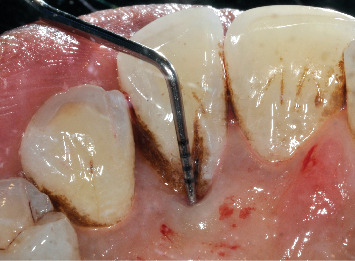
Palatal groove after nonsurgical therapy with probe inserted into the pocket.

The reevaluation at 3 months after nonsurgical therapy showed a significant improvement in all sites, resulting in pockets not exceeding 4 mm in depth and with no BOP and suppuration. Only one 5 mm pocket associated with BOP persisted at the developmental groove related to Tooth #2.2, which remained vital (Figure 3). Therefore, the decision was made to proceed with surgical intervention to eliminate the remaining pocket and improve plaque control by the patient, preventing recurrence and worsening in the indicated site.

Given the presence of the developmental groove as the likely main factor responsible for plaque accumulation and pocket formation, it was deemed necessary to intervene. Its presence would not only have caused the persistence but also the subsequent worsening of periodontitis in the site. However, it was evident that a simple access flap with debridement and cleaning of the involved root surfaces would not have been sufficient. Even with manual curettes and ultrasonic tips, cleaning within the invagination caused by the groove was challenging. Therefore, a decision was made to perform odontoplasty and radiculoplasty on the affected tooth.

**Figure 4:**
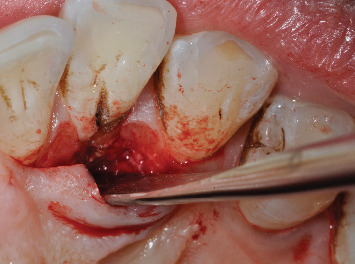
Palatal flap elevated after intrasulcular incision extended to mesial and distal teeth. Visible deposits on the root surface and in the groove.

**Figure 5:**
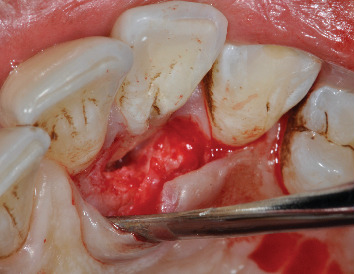
Root surface and groove after thorough cleaning and debridement.

**Figure 6:**
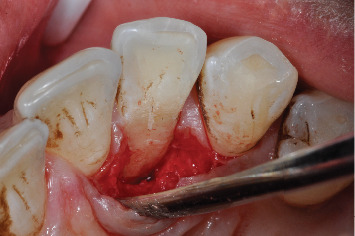
Palatal surface of the tooth after flattening the area where the palatal groove previously existed.

**Figure 7:**
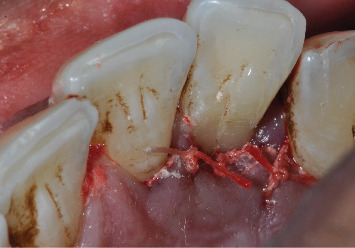
Flap sutured back in position.

An intrasulcular incision was made exclusively on the palatal side of the tooth, extending to the two teeth mesial and distal to it by vertically dissecting the two papillae (Figure 4). Once the flap was raised, the area was meticulously degranulated, and the root surface was cleaned, revealing the developmental groove in its entire length, which extended up to the middle third of the root without continuing further to the apex (Figure 5). The palatal surface of the tooth along which the groove was located was then flattened using a flame-shaped diamond bur and moved side to side until the groove disappeared, leaving a flat surface (Figure 6). After thorough irrigation with a saline solution, the flap was sutured back into position with two interrupted points on the papillae (Figure 7).

**Figure 8:**
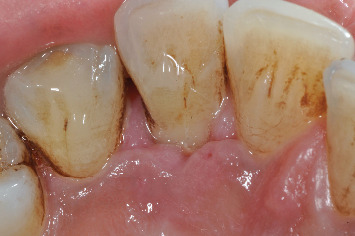
Healing at 2 weeks.

**Figure 9:**
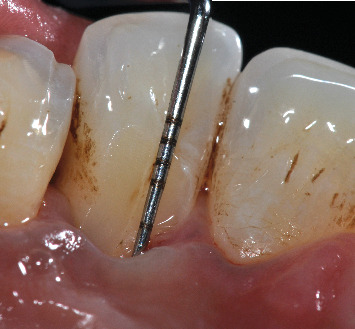
Healing at 6 months with reduced probing depth of 3 mm.

Two weeks after the surgery, soft tissue healing had occurred without any adverse events. The soft tissues appeared healthy, without swelling or bleeding, with two scars still visible corresponding to the incision lines on the papillae (Figure 8). At 6 months, the soft tissues were completely healed, and probing at the midpalatal point revealed a pocket depth of only 3 mm (Figure 9).

The patient, already very satisfied with the overall outcomes of the nonsurgical treatment received for his stage III grade C periodontitis, which led to a marked improvement in his perceived periodontal health, was also very pleased with the specific surgical intervention to address the developmental palatal groove on the lateral incisor. This satisfaction, expressed verbally, also translated into increased motivation and improved at-home plaque control.

## 3. Discussion

In this report, we present a case of a developmental groove on vital Tooth #2.2, occurring within the context of generalized Stage III Grade C periodontitis, causing localized worsening of periodontitis, successfully managed with access flap and odontoplasty after nonsurgical periodontal therapy.

The analysis of the periodontal charting, X-rays, and the patient's history led to a diagnosis of Grade C periodontitis. Under the old classification system, this case might have been classified as generalized aggressive periodontitis [23]. It was based on this diagnosis that systemic antibiotic therapy was incorporated into the nonsurgical treatment following the protocol by van Winkelhoff et al. [24, 25] This antibiotic regimen is indicated for rapidly progressing periodontitis, often associated with higher presence of *A. actinomycetemcomitans or Porphyromonas gingivalis*. Indeed, the nonsurgical therapy showed significant improvements in all sites except for the midpalatal site of the upper left lateral incisor. This further strengthens the initial hypothesis that the developmental groove was a local aggravating factor, diagnosed as a tooth-related modifying or predisposing local factor for periodontitis [4]. The palatal groove in the case described here can be classified as moderate according to Goon's classification [18] previously mentioned, as it does not involve the pulp and extends apically but not to the apex. According to Gu's classification [26], it is Type I, as it is a short groove not extending beyond the coronal third of the root.

In many cases, the developmental palatal groove can manifest in more severe forms, involving the dental pulp and thereby causing painful symptoms due to endodontic infection. In these cases, management becomes more complex, involving endodontic treatment of the tooth and the need to close the formed endoperiodontal communication, for example, using glass ionomer cement [27, 28] or the newest calcium silicate-based cements [29, 30]. If unrecognized or even misdiagnosed as a fracture, it can lead to tooth loss, both due to the uncontrolled progression of the pathology or iatrogenic causes (extraction), such as incorrect identification as a radiographic fracture [31]. However, diagnosis of a palatal groove poses significant challenges, as its symptoms might be mistaken for true periodontal disease or endodontic problem. Alternatively, the condition might be misclassified as an endodontic–periodontic problem. Consequently, a careful examination is essential for an accurate differential diagnosis. While the presence of a notch in the crown serves as a useful indicator, it can often be difficult to detect due to coverage by the gingival margin or plaque deposition. Furthermore, a localized periodontal deep pocket is a reliable indicator of a palatal groove. Additionally, the identification of a parapulpal line [6], a radiolucent line superimposed on or parallel to the root canal in periapical radiographs, assists in diagnosing a palatal groove.

Some cases in the literature report the treatment of a simple developmental groove (not involving the pulp) with debridement and root planing of the groove and placement of a membrane, with or without grafting with a biomaterial, to promote bone and periodontal ligament regeneration in the defect caused by the groove [32, 33]. However, this GTR option is possible only when the groove is not very deep; otherwise, there might be a risk of recurrence, especially when the groove has an evident continuity between the coronal portion of the tooth and the root. In our case, odontoplasty and radiculoplasty proved to be effective in treating the periodontitis associated with the groove. Nevertheless, it is important to emphasize that if the groove had extended beyond the middle third of the root to the apex of the tooth, this would not have been possible [6]. Performing radiculoplasty near the apex would undoubtedly have compromised the tooth's vitality.

Combined periodontal–endodontic lesions are frequently observed in cases exhibiting a progressive palatogingival groove. Successful treatment of these lesions requires addressing both conditions. Although complications due to palatogingival grooves are relatively rare, they may present a dilemma for general practitioner. Thus, a multidisciplinary approach is necessary. Inadequate or delayed treatment may lead to tooth extraction. The prognosis of a tooth with a palatogingival groove largely depends on the groove's location and type, as well as the accessibility of the defect. Therefore, precise diagnosis is crucial to preserving the tooth [26, 34].

## 4. Conclusions

The treatment of a site with residual attachment loss after nonsurgical periodontal therapy, associated with the presence of a palatal developmental groove extending from the crown's cervical region to the middle third of the root, has proven successful using a palatal access flap and odontoradiculoplasty, without showing any residual pockets 6 months after the procedure.

## 5. Take-Home Message

This case report underscores the importance of recognizing developmental grooves as significant factors in the progression of periodontitis. The effective management of such grooves through a combination of nonsurgical and surgical periodontal therapies can prevent further periodontal damage and improve patient outcomes.

## 6. Recommendations

Clinicians should prioritize early detection and accurate diagnosis of developmental grooves using advanced imaging techniques like CBCT. A multidisciplinary approach, involving periodontists and endodontists, is essential for comprehensive care. Tailored treatment plans, patient education on oral hygiene, and regular follow-up are crucial to ensuring long-term success and preventing the recurrence of periodontal issues.

## Data Availability

Data is available on request.
